# Communication and Inference of Intended Movement Direction during Human–Human Physical Interaction

**DOI:** 10.3389/fnbot.2017.00021

**Published:** 2017-04-13

**Authors:** Keivan Mojtahedi, Bryan Whitsell, Panagiotis Artemiadis, Marco Santello

**Affiliations:** ^1^Neural Control of Movement Laboratory, School of Biological and Health Systems Engineering, Arizona State University, Tempe, AZ, USA; ^2^Human Oriented Robotics and Control Laboratory, Mechanical and Aerospace Engineering, School for Engineering of Matter, Transport and Energy, Arizona State University, Tempe, AZ, USA

**Keywords:** human–human interaction, impedance, leader and follower, physical interaction, stiffness

## Abstract

Of particular interest to the neuroscience and robotics communities is the understanding of how two humans could physically collaborate to perform motor tasks such as holding a tool or moving it across locations. When two humans physically interact with each other, sensory consequences and motor outcomes are not entirely predictable as they also depend on the other agent’s actions. The sensory mechanisms involved in physical interactions are not well understood. The present study was designed (1) to quantify human–human physical interactions where one agent (“follower”) has to infer the intended or imagined—but not executed—direction of motion of another agent (“leader”) and (2) to reveal the underlying strategies used by the dyad. This study also aimed at verifying the extent to which visual feedback (VF) is necessary for communicating intended movement direction. We found that the control of leader on the relationship between force and motion was a critical factor in conveying his/her intended movement direction to the follower regardless of VF of the grasped handle or the arms. Interestingly, the dyad’s ability to communicate and infer movement direction with significant accuracy improved (>83%) after a relatively short amount of practice. These results indicate that the relationship between force and motion (interpreting as arm impedance modulation) may represent an important means for communicating intended movement direction between biological agents, as indicated by the modulation of this relationship to intended direction. Ongoing work is investigating the application of the present findings to optimize communication of high-level movement goals during physical interactions between biological and non-biological agents.

## Introduction

Collaboration, defined as the act of cooperation among multiple agents toward the attainment of a common goal, is one of the most sophisticated behaviors exhibited by biological organisms. Although cooperation is ubiquitous among a wide range of species ranging from ants to primates, the level of sophistication reached by humans in their ability to cooperate is unparalleled in the animal kingdom. Of particular interest to the neuroscience and robotics communities is the understanding of how humans collaborate to perform motor tasks.

Physical collaboration between two homologous biological agents, such as two humans holding a tool or moving it across locations, entails complex sensorimotor processes. Specifically, the problem of physically collaborating with another agent to perform a given motor task introduces control problems that go well beyond those encountered when controlling one’s own limb. For example, planning and execution of reaching or grasping movement are thought to occur through an internal model of the agent’s limb that allows prediction of the sensory consequences of the motor action (Johansson and Flanagan, 2009; Wolpert et al., 2011). Examples of such phenomena are the temporal coupling of grip and load forces associated with moving an object denoting anticipation of movement-related inertial forces (Flanagan and Wing, 1997), or the anticipatory control of torque prior to lifting an object with an asymmetrical center of mass (Salimi et al., 2003; Bursztyn and Flanagan, 2008; Fu et al., 2010, 2011; Fu and Santello, 2012; Mojtahedi et al., 2015). However, when two humans physically interact with each other, sensory consequences and motor outcomes are not entirely predictable as they also depend on the other agent’s actions. Therefore, the question arises as to how the central nervous system of each agent factors in the other agent’s actions when physically interacting with each other to perform a collaborative task. A better understanding of this problem can help developing biologically inspired controllers supporting human–robot physical interactions, e.g., exoskeletons used for neurorehabilitation or physical augmentation, and optimizing the way these interactions can be performed.

Physical interaction between humans and robots has been mainly investigated using the notion of mechanical impedance. Hogan (1985) first proposed robot impedance controllers as a way to guarantee stable and robust behavior of a robot that interacts with a human. Since then, a plethora of robot applications involving physical human–robot interaction use control of impedance, and in most cases, this is done to purposefully impose a specific dynamic behavior to the human agent. For example, the MIT-MANUS—used extensively for upper limb rehabilitation—uses the concept of impedance control in a back-drivable system to restrict the motion of the patient’s arm along a specific path, while the patient tries to reach a target *via* a manipulandum attached to his/her paretic arm (Krebs et al., 1998). For this scenario, impedance control is used to assist the human subject to reach a pre-defined target and imposes high resistive forces to motion that is not congruent with the desired trajectory.

The main objective of our study was to quantify the extent to which the human body (mainly upper limb) impedance can be used to infer intended movement direction of a cooperating agent in absence of other sensory cues (e.g., vision, hearing). Specifically, the present study sought to characterize the role of haptic information, which includes the relationship between force and displacement in a power exchange between two agents. We pursued this objective by quantifying human–human physical interactions where one agent (“follower”) was asked to infer the intended direction of motion of another agent (“leader”). In this design, the follower is trying to estimate the direction that the leader would allow them to move. Our interpretation of this interaction is that (1) the leader’s intended movement direction modulates this relationship in a direction-specific manner and (2) the follower can interpret this direction-specific modulation of this relationship to infer the leader’s intended movement direction. Note that the impedance in formal sense is quite complicated to measure due to the involvement of inputs/responses from both leader and follower who are physically coupled. So, even if the leader hypothetically modulated impedance to “instruct” the follower, the measure of leader’s impedance would not reflect the follower’s behavior as they both probe *and* react to the forces and motions. Briefly, we hypothesize that the emergent dyadic behavior (follower’s inference of leader’s intended direction) could be captured by the relationship between resultant force and displacement. Certainly, dyad’s arm impedances could affect this relationship, but certain aspect of dyad’s behavior interaction such as follower’s probing strategy could not be considered as impedance. Thus, the current study could only directly show and support how the relationship between force and displacement would change while we interpret the changes in the relationship as arm impedance modulation.

We also investigated the role of visual feedback (VF) in communicating intended directions *via* arm impedance modulation. We hypothesized that cooperating agents would be able to use arm impedance modulation to effectively communicate intended movement direction among cooperating agents. Previous studies have shown that humans can adapt to force fields during reaching tasks by modulating their arm impedance over time (Franklin et al., 2007; Wong et al., 2009). Therefore, we hypothesized that repeated exposure to the leader’s impedance would lead to a trial-by-trial modulation of arm impedance and improvement in follower’s ability to infer the leader’s intended direction. Finally, we hypothesized that haptic feedback would be sufficient to enable cooperating agents to accurately communicate intended movement direction through modulation of arm impedance.

## Materials and Methods

### Subjects

We tested 20 right-handed subjects (12 males, 8 females; age: 18–28 years). Hand dominance was self-reported. Subjects had no history or record of neurological disorders and were naïve to the purpose of the studies. Subjects gave informed written consent to participate in the experiments. The experimental protocols were approved by the Institutional Review Board at Arizona State University and were in accordance with the Declaration of Helsinki. Five pairs of subjects (dyads) were assigned to the experiment with VF, whereas the other five dyads were assigned to the experiment with no visual feedback (NVF).

### Experimental Apparatus

**Figure 1:**
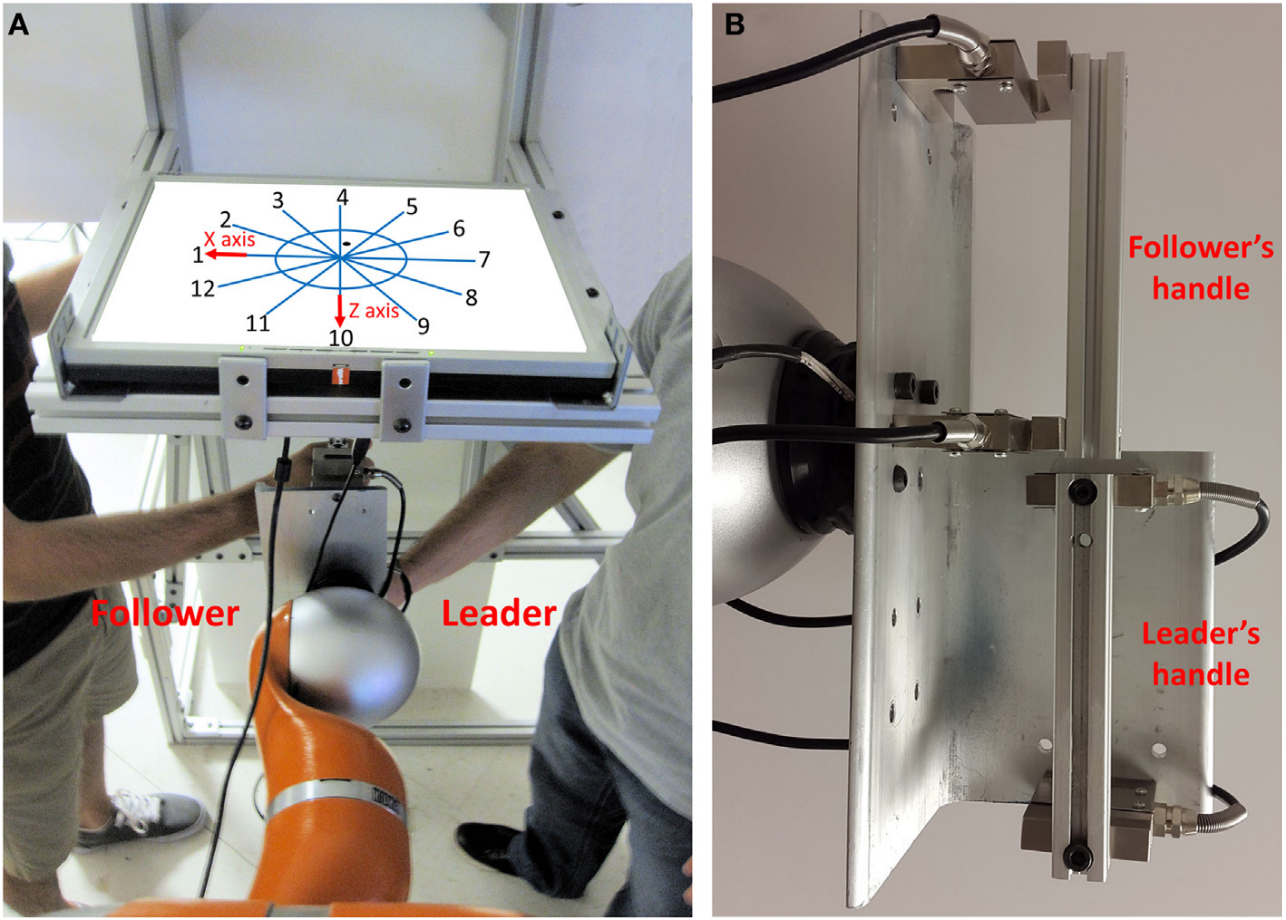
**Experimental apparatus**. **(A)** The robot arm constrains the movement of the grip device handle such that dyad can only move within the horizontal plane. The “leader” and “follower” grasped the lower and upper grip handles, respectively. Dyads were shown 12 direction lines and a circle on a computer screen. The cardinal directions are 1, 4, 7, and 10. The dot position on the screen was colocated with the position of the handle. **(B)** The follower and leader grasp upper and lower handles, respectively.

Each dyad was shown 12 lines oriented 30° apart from each other denoting movement direction and a circle (5-cm radius) on a computer screen (Figure 1). A number (1–12) was displayed at the outer end of each line. In the VF experiment, the dyad saw a dot on the screen. The dot position was colocated with the position of the handle the two subjects were holding and was located underneath the screen. The dot displayed on the monitor moved the same amount as the handle (ratio 1:1). The handle movement was constrained by the robot arm in the horizontal plane. The screen prevented the dyad from seeing the arm configuration of the other agent and the grip handle. In the NVF experiment, the dyad could not see the dot position but could still see the direction lines and circle.

For both experiments, we used an anthropomorphic 7-degree-of-freedom robot arm (LWR4+, KUKA) with the associated KRC robot controller and the KUKA’s Fast Research Interface. We used two load cells (Model: 3140-500 kg, precision: 0.02% FS, one-axis force sensor) embedded in the grip device to measure the resultant forces of the dyad in *x*- and *z*-axis (Figure 1).

### Experimental Tasks

One subject was designated as the “leader,” whereas the other was designated as the “follower.” At the start of the trial, the handle and corresponding dot displayed on the screen were positioned in the center of the circle. For each trial, the experimenter showed a specific number on a sheet to only the leader. This number was one of the 12 possible movement directions, which we will refer to as the “intended direction of movement” for that trial. The leader was instructed that his/her goal was to plan the movement in the direction that was shown to them by the experimenter while keeping the object as close as possible to the center of the circle (Figure 1). Therefore, leader thought about performing a movement rather than executing it in the direction assigned by the experimenter. The follower was instructed that his/her goal was to infer the leader’s intended direction of movement. The follower was also instructed that he/she could move the grip handle as he/she desired, but that he/she had to stay within the circle. The leader was instructed to react to the forces and motion of the follower while preserving the intention to move in a given intended direction. Thus, the leader tried to hold the handle in the middle of the work space and resisted all motion. The follower explored the space to infer the intended direction of leader. Whenever the position of the grip handle and the corresponding dot moved out of the circle, the color of circle and direction lines changed from blue to red to signal that the trial had to be stopped and repeated. Therefore, both groups received VF of the error, i.e., they were shown when the grip handle crossed the boundaries of the circular workspace. The subject pairs in the VF group never moved out of range. For the NVF group, the handle moved out of range only on four trials performed by three subject pairs (0.95% of all trials across five subject pairs in NVF). The grip handle range of motion was not physically constrained. After performing each trial, the follower was asked to write the number of the inferred direction on an answer sheet. During the whole experiment, neither the follower nor the leader received any feedback about his/her performances from experimenter, nor was the leader informed about the follower’s performance by the follower or the experimenter. Verbal communication between the subjects, as well as between the subjects and the experimenter, was not allowed before, during, or after the trial.

The role of each subject in the dyad can therefore be described as follows.

The “leader” was asked to

- Plan his/her intended direction (1 out of 12).
- Sense the follower’s applied force direction.
- React to the follower’s forces by maintaining the handle as still as possible at the center of the circle while preserving the intention to move in the instructed direction.

The “follower” was asked to

- Apply forces to infer the leader’s planned movement direction while remaining within the circle (5 cm radius).
- Sense the leader’s reaction to his/her own forces.
- Infer the planned direction and write it in the answer sheet.

Subjects were asked to keep their right hand close to the grip handles and wait for a “go” signal. As soon as they heard the “go” signal, they were asked to grasp the handle and started to interact with each other. Subjects initially performed 24 trials (2 repetitions per directions; Trial: 1–24) to reach a plateau in the performance, e.g., correct inference of the leader’s intended movement direction. Pilot data had revealed that this number of trials had been found to be sufficient for familiarization purposes. Then, subjects continued to perform 60 more trials (5 repetitions per direction; Trial: 25–84). The order of directions was randomized in both Trial: 1–24 and Trial: 25–84. We used different randomized order across dyads. Each trial lasted 30 s. The same instructions were given to the groups with and without VF.

At the beginning of each trial, the arm posture was inspected by the experimenter to ensure the same posture would be used across trials. Handle position was always located on the sagittal plane, and the trunk was as close as possible to the frame to prevent both subjects from viewing their arm configuration. The experimenter also verified that subjects kept their gaze on the monitor on each trial.

### Data Recording, Processing, and Experimental Variables

The robot was used to restrict motion of the fixture to the horizontal plane, prevent rotation of the fixture, and record the position of the grip handle during the experiment. We synchronized collection of position and force data. Position and force data were recorded at a sampling rate of 100 Hz and run through a fifth-order Butterworth low-pass filter (cutoff frequency: 30 Hz). The first-time derivative of force or position data was also low-pass filtered with cutoff frequency of 15 Hz.

### Percentage of Inferences

**Figure 2:**
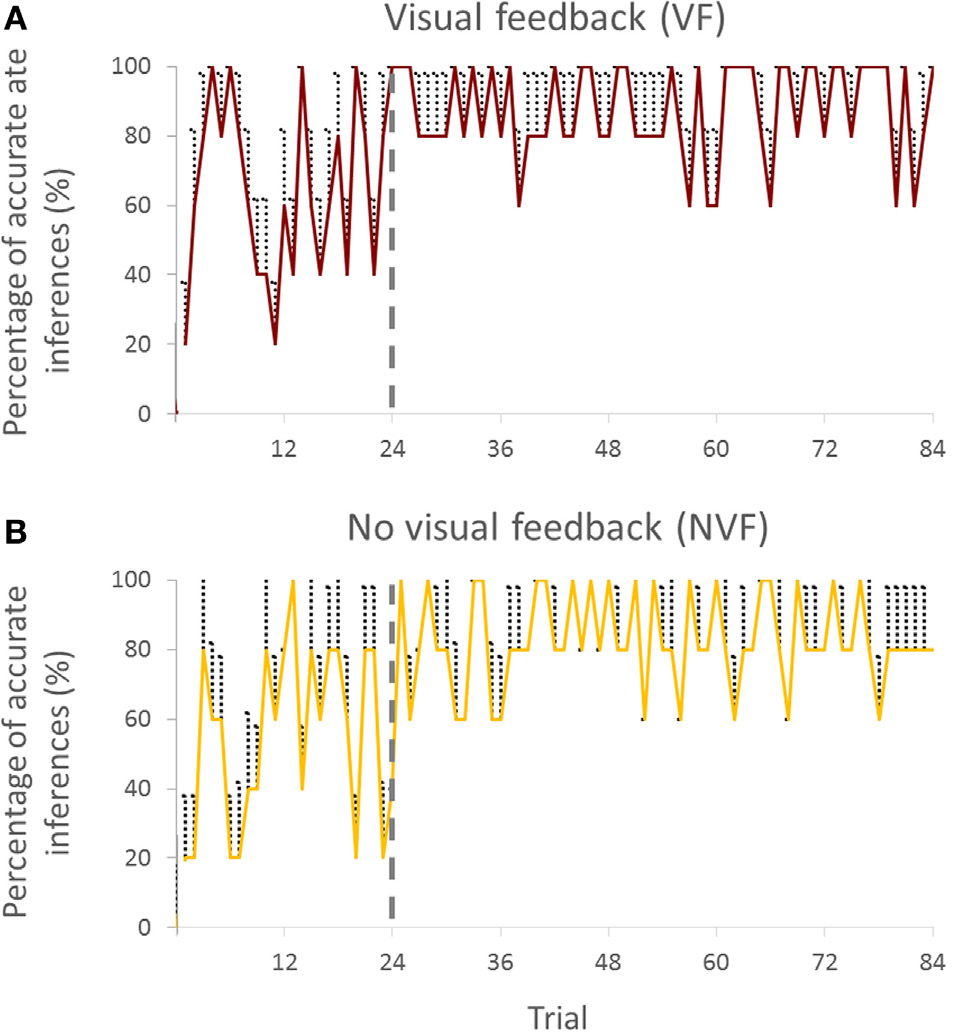
**Percentage of accurate inferences of follower across trials for both visual feedback (A) and no visual feedback (B) groups (all subjects)**. Vertical bars denote SEMs.

The dyad’s goal was to minimize the error between the leader’s intended direction and the follower’s inference. Therefore, all the metrics were defined based on this task requirement. To quantify the extent to which the follower could correctly infer the leader’s intended movement direction, we computed the percentage of accurate inferences (PAI) by each follower relative to the total number of trials based on his/her responses in the answer sheet. The follower’s error direction with respect to the leader’s intended direction was defined as the difference between leader’s intended direction and follower’s response. This error direction could be any number ranging between −6 and +5. An accurate inference of the follower would correspond to a difference of 0, whereas non-zero differences would denote inaccurate inferences. Positive sign of error direction (with respect to the leader’s intended direction) indicated counterclockwise difference between the leader’s intended direction and the follower’s response (Figure 1). One error direction with respect to intended direction is equivalent to 30° (Figures 1 and 2).

In Figure 1, we define the directions 1, 4, 7, and 10 as *cardinal* directions and all other directions as *non-cardinal*. This distinction was motivated by the fact that accurate inferences of the leader’s intended movement direction differed across cardinal versus non-cardinal directions (see Statistical Analysis).

### Force–Displacement Relationship

In the present work, the term impedance denotes to the effect of voluntary muscle activations of mainly the upper limb to the limb dynamics. Those dynamics largely affected by the muscle activations include both the stiffness and the damping characteristics of the arm, which are only apparent when there exists force interaction with the environment, in the current case the follower. In the dyadic interaction, the resultant force denotes to the net force which both leader and follower together generated on the handles. In the current experiment, the robot is entirely passive because the friction and damping effects of the robot are feed-forwarded to the joints by a torque controller, and therefore, their magnitudes are negligible (close to 0; the robot inertia and damping are by default compensated through the built-in impedance controller of the KUKA arm). The inertia effects are also negligible due to the low acceleration in the handle motion. The average of absolute acceleration of the handle was 16.02 (±0.35) cm/s^2^ (1.63 ± 0.04% of gravity acceleration). Thus, the damping and inertia effects are minimum relative to the stiffness effects due to the low velocity and low acceleration, respectively, of the handle motion. Therefore, consistent with the previous work (Mussa-Ivaldi et al., 1985; Formica et al., 2012), we assume that the total change in force [Δ*F*(*n*)] is primarily caused by the stiffness of the subjects interacting with each other.

In the present work, the estimation of the stiffness depends on the follower’s exploration of the workspace, which is very different from the systematic exploration of an equal number of movement directions as tested in previous work (Perreault et al., 2001; Krebs et al., 2003). So, we had to use a different approach to quantify the relationship between force and motion due to limitations of applying a conventional approach to estimate stiffness. We used Eq. 2 to calculate the force–displacement relationship (|*k*|):

(1) $$F(n)=\sqrt{F_{x}^{2}(n)+F_{z}^{2}(n)};\;U(n)=\sqrt{U_{x}^{2}(n)+U_{z}^{2}(n)}$$

(2) $$k(n)=\frac{\Delta F(n)}{\Delta U(n)}=\frac{F(n)-F(n-1)}{U(n)-U(n-1)}$$

Equation 1 describes how to calculate the force [*F*(*n*)] and displacement [*U*(*n*)] magnitudes for each time point (*n*). As we see later in Figure 5, the follower did not explore all the points in the circle with 5-cm radius; and it illustrates that the magnitudes of force and displacement are changing reversely regardless of their directions or vector properties, e.g., small forces and large displacements are along the intended direction, while we have large forces and small displacements for other directions (Figures 5A,B). Therefore, it seems that considering only magnitudes is enough to discriminate the relationship between force and displacement across directions. So, the *k* in Eq. 2 should be calculated on the points visited by the follower. To avoid the canceling effect of *k* value due to positive and negative *k* values (according to movement direction relative to start location), we computed absolute value of *k* [|*k*(*n*)|]. For example, if the follower pulled the handle in direction 1 for 3 cm distance, this creates positive values of *k* in Eq. 2. By contrast, when the leader pulls back the handle to the center to maintain the handle at the center, this creates negative values of *k* in Eq. 2.

As |*k*| value was associated with a specific position point within the circle (Figure 1), we averaged |*k*(*n*)| over time to obtain the best value of the force–displacement relationship for each visited point. We gave |*k*| the value of 0 to the points that were not visited. If the position of the grip handle did not change relative to its starting position (center of the circle), we assigned the maximum value of |*k*| of that trial (infinite) to the |*k*| at that position. The denominator is equal to 0 while the handle position does not change. So, we consider the |*k*| as infinite (the maximum |*k*| which is recorded when they did move the handle in that trial).

The position resolution in the horizontal workspace plane was 1 mm^2^. We calculated the average of non-zero values of |*k*| for all the position points within each 1 mm^2^ and assigned that |*k*| value to that square. Therefore, each square in the horizontal plane was assigned a specific |*k*| value. By doing so, the average |*k*| associated with each direction could be obtained by calculating the average of non-zero values of |*k*| of the squares located in that direction (within ±15° of each direction). This procedure led to the extraction of 12 |*k*| values, 1 for each of the 12 movement directions with respect to the leader’s intended direction (|*k*|*_i_*_,Average_; *i*: −6 to +5; *i* is movement direction with respect to intended direction). First, we calculated the average |*k*| across all movement directions (|*k*|_Average_; Eq. 3). Second, we normalized the |*k*| values (|*k*|*_i_*_,Normalized_; *i*: −6 to +5; Eq. 5) based on the maximum of the average |*k*| values in all directions for each trial (|*k*|_Max_; Eq. 4) to remove differences in |*k*| across dyads.

(3) $${\left|k\right|}_{\text{Average}}=\frac{1}{12}\sum_{i=-6}^{5}{\left|k\right|}_{i,\text{Average}}$$

(4) $${\left|k\right|}_{\text{Max}}=\text{max}\left({\left|k\right|}_{-6,\text{Average}},{\left|k\right|}_{-5,\text{Average}},\ldots ,{\left|k\right|}_{+5,\text{Average}}\right)$$

(5) $${\left|k\right|}_{i,\text{Normalized}}=\frac{{\left|k\right|}_{i,\text{Average}}}{{\left|k\right|}_{\text{Max}}}$$

Briefly, we had 100 × 100 points (|*k*| estimates). We assigned a 0 value to |*k*| at the points that were not visited by the handle. For the visited points, we obtained the average value of estimated |*k*| across time samples to capture the behavior of both leader and follower at that point. We then calculated the spatial average of |*k*| values (non-zero values) within ±15° of each direction to obtain average |*k*| of that direction. Although the measure of |*k*| is not formal stiffness or impedance, it is suitable for capturing the relationship between force and motion of a dyad in our paradigm, which may imply aspects of dyad’s modulation of stiffness/impedance.

### Statistical Analysis

#### Inference of Intended Movement Direction

We chose to break down the trials by 24 for the first block and 60 trials for the second block. Analysis of pilot data revealed that the accuracy of predicting the intended movement direction reached a maximum and converged to a steady state after the first 24 trials. The results of the current study also confirmed these pilot observations (Figures 2A,B).

**Figure 3:**
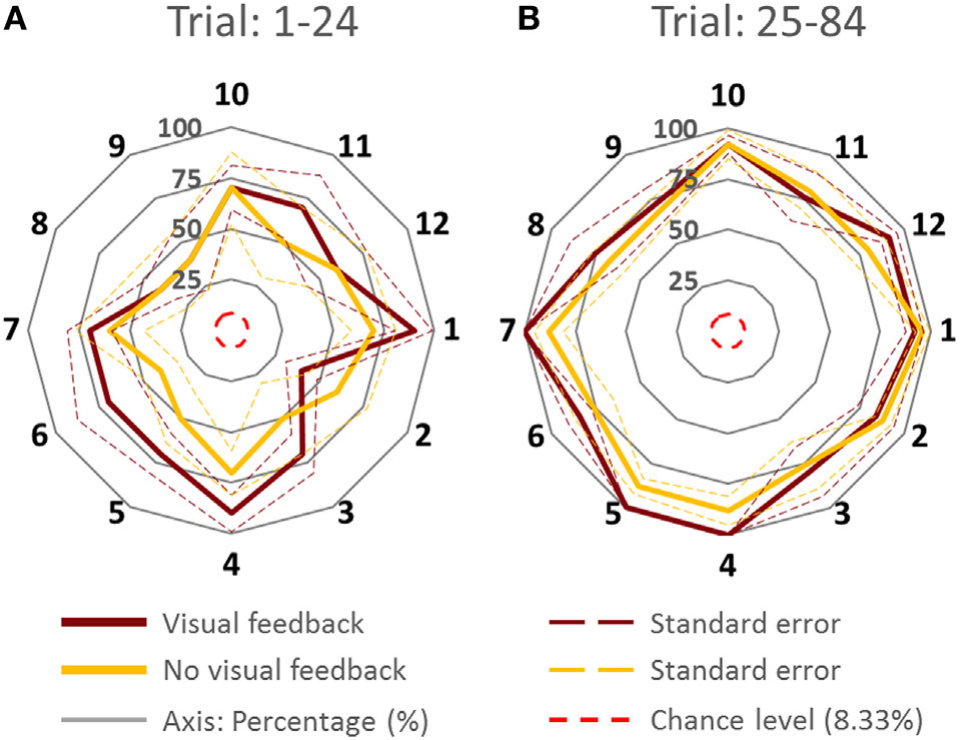
**Percentage of accurate inferences of follower for each leader’s intended movement direction computed (across all subjects)**. **(A)** Trial: 1–24. **(B)** Trial 25–84. Data for each direction are means averaged across all subjects. The cardinal directions are 1, 4, 7, and 10. The dash lines represent the SE. The four concentric gray circles represent the ring axes of percentage. 100% is the biggest ring, while 0% is a dot in the center which is not shown.

To assess whether PAI was sensitive to the leader’s intended direction along the cardinal directions and non-cardinal directions, we performed analysis of variance (ANOVA) with repeated measures on PAI with two within-subject factors (Trial; trials 1–24 and trials 25–84; two levels, Direction; cardinal and non-cardinal directions; two levels) and one between-subject factor (Group; VF and NVF groups, two levels; see Figure 3).

#### Force–Displacement Relationship Is Modulated across Trials for the VF Group

We performed ANOVA with repeated measures on the average |*k*| of all directions, |*k*|_Average_, with one within-subject factor (Trial; two levels). We also performed ANOVA with repeated measures on the normalized |*k*|, |*k*|*_i_*_,Normalized_, with one within-subject factor (Direction; direction −6 to +5; 12 levels; Figures 6C,D). Note that |*k*| normalization was performed to only remove the strength differences between subjects.

Comparisons of interest for statistically significant differences (*p* = 0.05) were further analyzed using *post hoc* tests with Bonferroni’s corrections. We conducted the normality and sphericity tests and statistical models were valid. Statistical analysis was performed using IBM Sciences Statistical Package for the Social Statistics.

## Results

### PAI of Follower

Percentage of accurate inference analysis was divided into three sections. First, we report the evolution of PAI over trials. Second, we investigate the effects of Group, Trial, and Direction on PAI by keeping the original direction in order to assess the effect of cardinal directions on PAI. Third, we assess PAI without regarding the effect of direction to assess how both groups performed when we had a common reference (i.e., 0 error direction).

#### PAI Analysis across Trials

Visual inspection of the trial-to-trial fluctuations of the PAI revealed that performance was more variable in the early trials (1–24). To minimize the effect of large random trial-to-trial PAI fluctuations in these early trials, for statistical purposes, we averaged PAI across a variable number of trials. We found that averaging PAI across 3, 4, 6, 8, and 12 trials gave approximately the same result, i.e., PAI stopped improving after the first 24 trials.

Percentage of accurate inferences improved in both VF and NVF groups (Figures 2A,B, respectively). In the beginning, both groups could not perform consistently above 60% of PAI. However, after approximately 24 trials, both groups reached a steady-state performance.

We analyzed the time it took followers to report inferred leader’s intended direction. When VF was available, followers reported the follower’s intended direction within 29.6 ± 0.2 s, whereas the response time was slightly shorter (27.4 ± 1.1 s) when VF was not available.

#### Cardinal versus Non-Cardinal Directions

Figure 3 shows PAI for all directions. We compared the PAI associated with the leader’s intended movement in the cardinal directions (1, 4, 7, and 10; Figure 1) versus non-cardinal directions. Although VF did not affect PAI [no main effect of Group; *F*(1,8) = 0.697, *p* = 0.428], PAI was significantly different as a function of Trial [*F*(1,8) = 28.891, *p* = 0.001, η^2^ = 0.78] and movement direction [*F*(1,8) = 7.254, *p* = 0.027, η^2^ = 0.47]. No significant interactions were found (all *p* values >0.320). As found earlier across all movement directions, PAI of Trial: 25–84 was significantly larger than Trial: 1–24 (*p* = 0.001). For the experimental trials, PAI associated with the leader’s intended movement along the cardinal directions was significantly larger than along non-cardinal directions (93.5 and 80.5%, respectively; *p* = 0.027).

#### VF versus NVF

**Figure 4:**
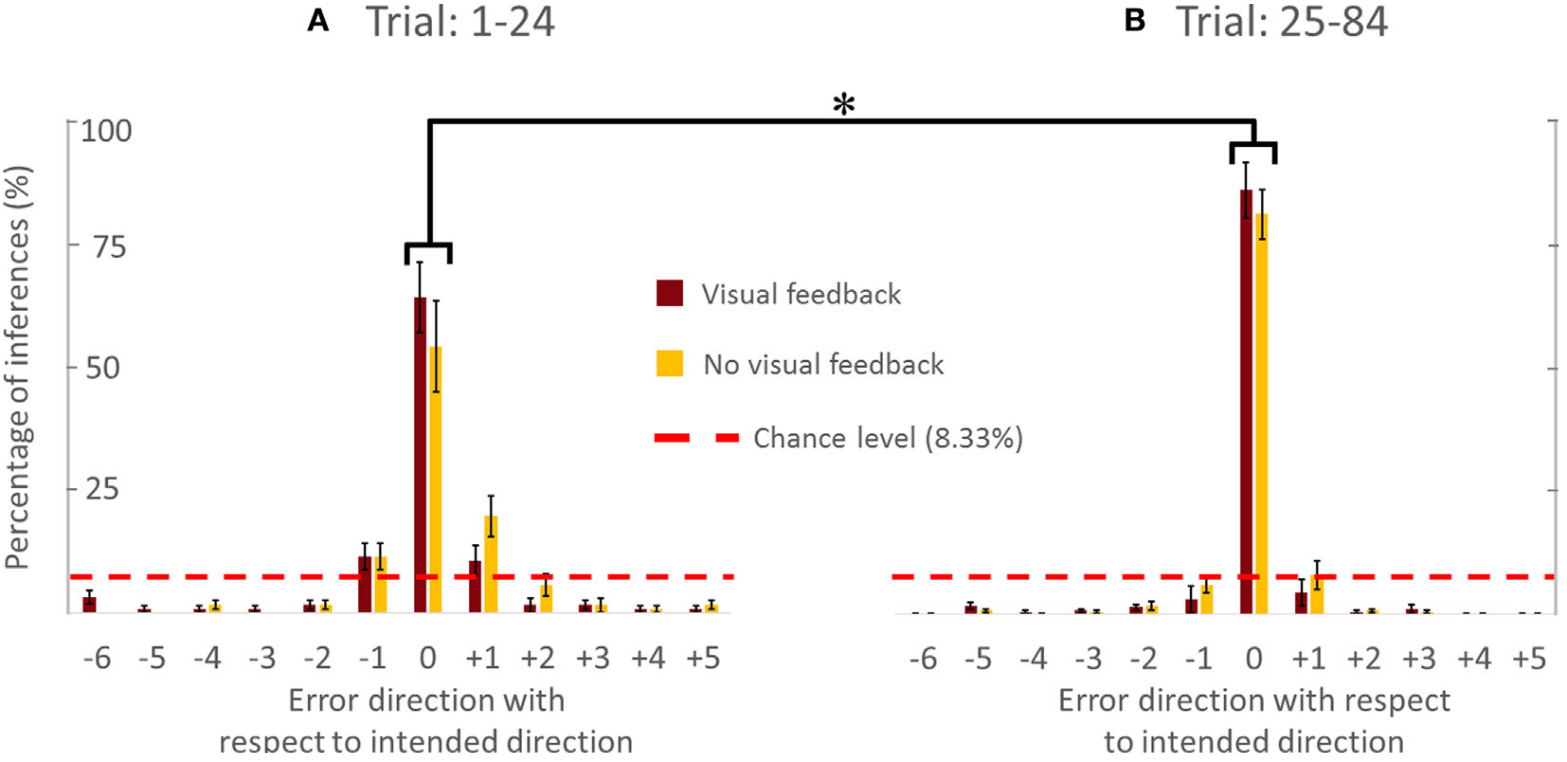
**Percentage of accurate and inaccurate inferences of follower (all subjects)**. **(A)** Trial 1–24. **(B)** Trial 25–84. Data are means averaged across all subjects. Vertical bars denote SEMs. Asterisks indicate a statistically significant difference (*p* < 0.001). Percentage of accurate inferences is the percentage value of 0 error direction with respect to intended direction. One error direction with respect to intended direction is topographically equivalent to 30°.

Figure 4 showed the PAI of follower for the VF and NVF groups computed from Trial: 1–24 and Trial: 25–84 (Figures 4A,B, respectively). PAI was well above chance level (equivalent to 1 out of 12 possible directions, i.e., 8.33%). After 24 trials, PAI values were 87.33 and 83.33% when performed with and without VF, respectively. If we assume that ±1 error direction with respect to intended direction is a negligible performance error (±30°), the combined PAI were 94.67 and 96.33% for trials performed with and without VF, respectively.

The availability of VF did not significantly affect PAI [no main effect of Group; *F*(1,8) = 0.535, *p* = 0.485]. However, we found statistically significant differences in PAI as a function of Trial [*F*(1,8) = 30.444, *p* = 0.001, η^2^ = 0.79], but no significant interaction between Group and Trial [*F*(1,8) = 0.309, *p* = 0.594]. We found that PAI from Trial: 25–84 was significantly larger than from Trial: 1–24 (Figure 4; *p* = 0.001). Note that we reported the effect size (partial-eta squared) as a measure of magnitude of our effect. The effect size of learning was quite large (η^2^ = 0.79). This indicates that the significance of the result was unlikely to be marginal, for example, large variation within one subject could have driven the result. Therefore, we were confident that our sample size (five subjects per group) was adequate. The results (effect sizes) were highly consistent among the 5 subjects within each group, and—most importantly—highly consistent across the 10 subjects across both groups.

In summary, the follower’s ability to infer the leader’s intended movement direction was insensitive to whether the follower could view the position of the dot on the screen or not. Furthermore, PAI improved with practice, implying that the follower and leader gradually adapted to each other’s actions to communicate and collaborate with each other. Specifically, the follower learned to infer the leader’s intended movement direction, of the leader, while the leader learned how to react to the follower’s forces. Finally, the follower was more accurate in inferring the leader’s intended movement direction for cardinal than non-cardinal directions.

### Force–Displacement Relationship Analysis

We first present one representative trial from a dyad performing our task with VF, followed by analysis of |*k*| adaptation for the VF group. We present the force–displacement relationship analysis for only VF group because they had the reference point of the center of circle. This allowed us to perform force–displacement relationship analysis relative to this reference point.

#### Representative VF Trial

**Figure 5:**
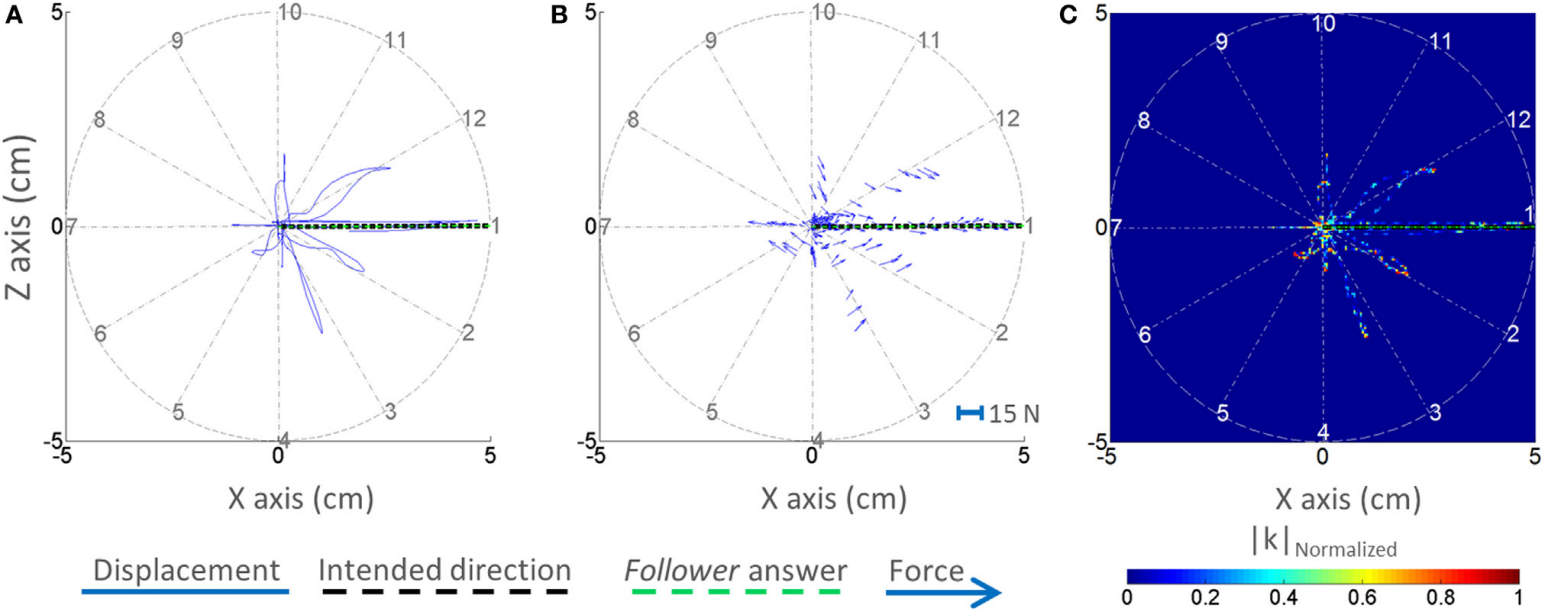
**Displacement, forces, and normalized |*k*|–position profiles**. One representative trial is shown for the dyad from the visual feedback group. The displacement–position profile is shown in panel **(A)**. The force–position profile (quiver plot) is shown in panel **(B)**. The normalized |*k*|–position profile is shown in panel **(C)**. The selected trial is representative of correct response.

Figure 5 shows the displacement–position profile (Figure 5A), force–position profile (Figure 5B), and normalized |*k*|–position profile (Figure 5C) of a sample trial of a VF dyad. The displacement–position profile for the VF group reveals that the dyad performed the task as instructed, i.e., within the boundaries and close to the center of the circular workspace. Note that the dyad exhibited larger handle displacement along the leader’s intended direction (Figure 5A). With regard to the force–position profile of the VF group, the leader could generate a reasonable force field (impedance field) for each direction as if the resultant force tended to be directed toward the leader’s intended direction at each position (Figure 5B). Visual examination of the normalized |*k*|–position profile of the VF group reveals that the dyad exhibited lower |*k*| in the intended leader’s movement direction (Figure 5C).

#### Force–Displacement Relationship Analysis: Dyads with VF

**Figure 6:**
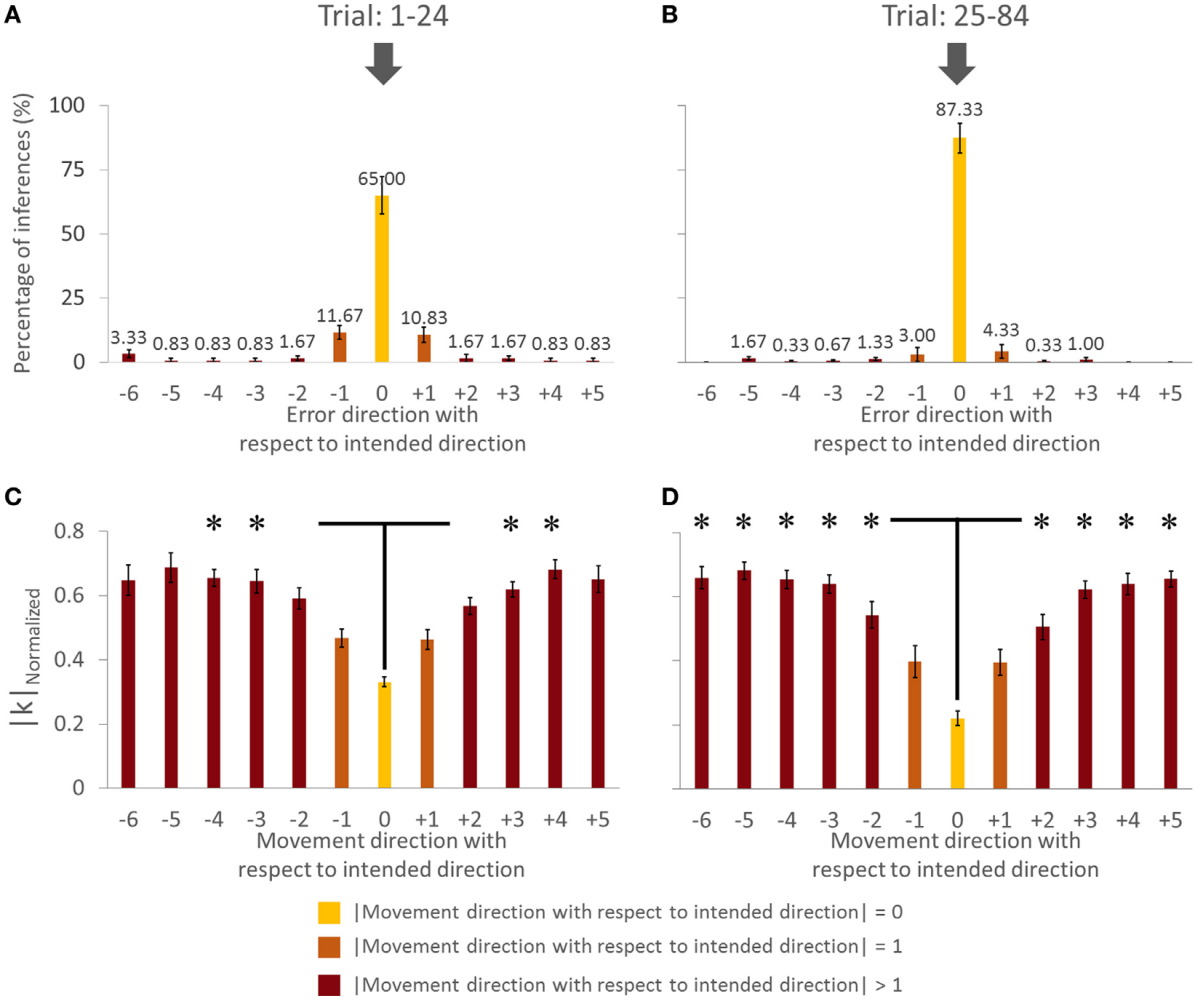
**Force–displacement relationship analysis of group with visual feedback (all subjects)**. **(A,C)** Trial: 1–24. **(B,D)** Trial: 25–84. Asterisks in panels **(C,D)** indicate a statistically significant difference of pairwise comparison between 0 (yellow bar) and other (dark brown bar) movement direction with respect to intended direction (*p* < 0.05). Data in panels **(A–D)** are means of values averaged across all subjects. Vertical bars denote SEMs.

To elucidate the force–displacement relationship analysis in the VF group, we compared the |*k*| measured during the dyad interaction during trials 1–24 and trials 25–84. We captured the evolution of normalized |*k*| (|*k*|_Normalized_) across two blocks of trials (1–24 and 25–84; Figures 6C,D). We then performed pairwise comparisons of |*k*|_Normalized_ within each block to investigate how dyads selectively generated |*k*|_Normalized_ across different directions with respect to the leader’s intended movement direction.

**Figure 7:**
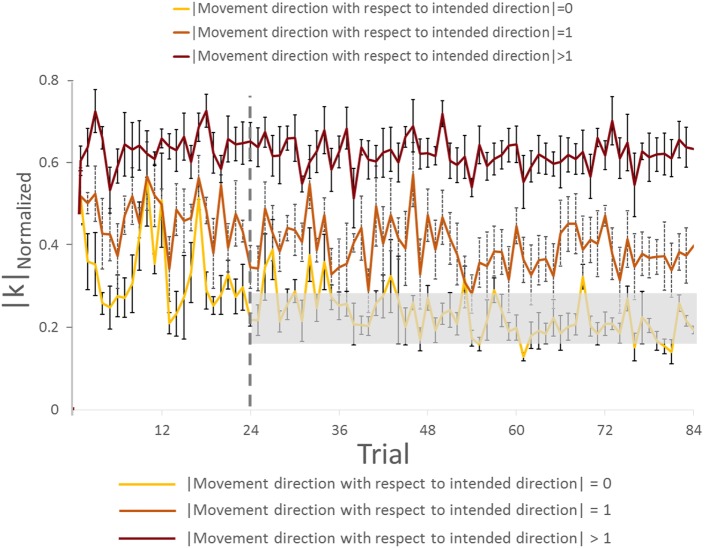
**Normalized |*k*| analysis of group with visual feedback across trials (all subjects)**. The gray rectangle box represents the range of normalized |*k*| (mean value ±3 × SE) on movement direction with respect to intended direction. Vertical bars denote SEMs.

We found a main effect of Direction in |*k*|_Normalized_ for both trials [in Trial: 1–24, *F*(11,44) = 15.182, *p* = 0.001, η^2^ = 0.81; in Trial: 25–84, *F*(11,44) = 37.058, *p* = 0.001; η^2^ = 0.90]. For the Trial: 1–24, we found no significant difference in |*k*|_Normalized_ on pairwise comparisons between the intended movement direction (0, yellow bar) and directions of ±1 (orange bars), −6, ±5, and ±2 (dark brown bars, Figure 6C; all *p* > 0.05). However, there was significant difference in |*k*|_Normalized_ between intended direction and adjacent directions of ±3 and ±4. Similarly for Trial: 25–84, no significant differences were found when comparing |*k*|_Normalized_ at the leader’s intended movement direction and adjacent directions (±1; orange bars). However, we found significant difference in |*k*|_Normalized_ on pairwise comparisons between the intended movement direction (0, yellow bar) and all other directions except ±1 (dark brown bars, Figure 6D; all *p* > 0.05). Figure 7 illustrates how |*k*|_Normalized_ of movement direction with respect to intended direction changed across trials. Figure 7 shows that the variations in |*k*|_Normalized_ of movement direction with respect to intended direction of 0 (yellow line in Figure 7) were gradually settled in the gray box over the trials and also became more discriminable from |*k*|_Normalized_ of movement direction with respect to intended direction of 1 (orange line in Figure 7). Figure 7 shows how the force–displacement relationship in dyadic interaction evolves across trials, and this might imply that dyad learns to modulate their stiffness to perform the task.

To further quantify the effect of trial (practice) on force–displacement relationship modulation, we compared the average |*k*| of all directions (|*k*|_Average_) across two blocks of trials (1–24 and 25–84). Statistical results showed that |*k*|_Average_ did not change significantly across Trial (*p* = 0.447).

These results indicate that, following early exposure to our task, the dyad learned to modulate force–displacement relationship across movement directions. Importantly, the dyads’ |*k*| became minimum at the leader’s intended movement direction, even though the average |*k*| did not change significantly.

## Discussion

The primary goal of our study was to quantify the extent to which human body impedance can be used to infer intended movement direction of a cooperating agent. We found that the success in conveying inferring intended direction of motion between two agents was correlated with the control of leader’s impedance as a function of the follower’s direction of motion. Hence, we were able to show that the leader was conveying the information of intended direction to the follower by controlling his/her impedance at the object they were interacting with. Therefore, our results may imply that two cooperating agents could use arm stiffness modulation during physical interaction as a viable means of communication of intended movement direction. We discuss these results in relation to previous work, potential sensorimotor mechanisms, and applications of the proposed framework to human–robot interactions.

### Effect of Practice on Accuracy of Movement Direction Inferences and Force–Displacement Relationship

A moderate amount of practice (1–24 trials) led the followers to a significantly greater accuracy of inferences of the leader’s intended movement direction (Figures 2 and 4). This result indicates that subjects might have needed some practice to gauge and interpret each other’s physical response. Nevertheless, the small number of trials leading to a very high level of accurate inferences (>83%) also suggests that humans (a) can maintain the high level of accurate responses after 24 trials (Figures 2 and 4), which might imply that within 24 trials the dyad was already specialized for modulating arm stiffness, (b) are very sensitive to the directional tuning of arm impedance (see force–displacement relationship analysis below; Figure 6), and (c) can therefore learn fairly quickly to correctly interpret such directional tuning (Figure 7).

The adaptation of force–displacement relationship (|*k*|) as function of intended movement direction evolved gradually (Figures 6C,D). Gradual discrimination of normalized |*k*| across different direction of motion (Figure 7) implies that dyads could convey the intended direction of motion by modulating and perceiving normalized |*k*| associated with the physical interaction. We interpret these data as follows: after performing 24 trials, the leader selectively generated less |*k*| in his/her intended direction in response to follower’s force perturbations. Therefore, we interpret this finding as evidence that control of the leader’s stiffness might be a critical factor in conveying the intended direction to the follower regardless of VF of the handle or arms. Our results also indicate that subjects learn this strategy by experiencing our task for the 12 directions. It is conceivable that exposure to fewer movement directions might result in faster learning across trials, or shorter exploration duration within each trial.

### Sensorimotor Integration Mechanisms for Movement Direction Inference

Visual feedback of the movement of the shared handle did not affect the extent to which followers correctly inferred the leader’s intended movement direction. This result suggests that haptic feedback elicited by physical interaction is sufficient to extract intended movement direction from the perceived force–displacement relationship (Figures 2 and 4). VF of movement trajectory was not necessary also in tasks performed by individual subjects requiring adaptation to stable or unstable dynamics (Franklin et al., 2007). Furthermore, final adaptation was similar with and without VF even when the learning signals (proprioception and vision versus only proprioception) were different (Franklin et al., 2007). Another study found that visual information of the movement trajectory alone might not be sufficient to modulate limb stiffness in response to an unstable elastic force field applied to the limb (Wong et al., 2009). Specifically, such adaptation might rely on somatosensory feedback to a greater extent than vision because of a direct relationship with perturbing forces. In reaching tasks, visual perturbations (manipulation of the cursor position) did not result in stiffness modulation, whereas force perturbation in elastic force field caused a significant increase in stiffness (Wong et al., 2009).

Movement kinematics appears to be sensitive to whether VF is available or not during adaptation of movement trajectories. Specifically, the movement profiles were significantly more linear when VF was available in “no force” and “velocity force” fields. However, the linearity did not change for visual and no visual conditions in a position force field (Franklin et al., 2007). The current study found different movement profiles of net displacement during the physical interaction between VF and NVF groups. However, even with this difference, the subjects learned the task and performed equally well in later trials. We should note that these results do not rule out a role of VF in our physical interaction task, but rather point to the fact that haptic feedback alone was accurate enough to enable correct inference of intended movement direction.

Subjects’ ability to use non-visual feedback to estimate human body (mainly upper limb) stiffness and infer intended movement direction likely arises from their ability to integrate sensory feedback about movement and force. Specifically, based on the definition of stiffness, movement direction associated with low stiffness would result in a larger displacement due to smaller force and smaller displacement due to larger force for high stiffness. Our focus on stiffness incorporates this relation between force and displacement, and our interpretation about stiffness as a means of communication includes the use of position sensing for this purpose. Specifically, we propose that position and force sensing combined was involved in the estimation of intended movement direction (see Supplementary Material; Figures S1 and S3 in Supplementary Material). As impedance cannot be sensed by a specific type of sensory receptor, impedance estimation would have to rely on integrating estimation of muscle length and force, each of which is mediated by distinct mechanoreceptors (muscle spindles and Golgi tendon organs, respectively). In Figure S3 in Supplementary Material, the logistic regression analysis is in favor of the proposed notion that force–displacement relationship (|*k*|) is a better predictor of PAI than either average resultant force or maximum displacement alone at intended direction. This proposition is also consistent with experimental evidence showing that subjects estimate object stiffness by differentially weighing feedback information provided by muscle length and force receptors (Mugge et al., 2009). Since the leader was never required to execute a voluntary movement but just to “plan” (but not execute) a movement in a prescribed direction, it is conceivable that motor cortical areas involved with upper limb control were activated, as shown by literature on motor imagery (e.g., Vogt et al., 2013; Eaves et al., 2016; Hanakawa, 2016).

### Impedance-Based Communication in Human–Human and Human–Robot Interactions

Human arm impedance control has received increased attention during the last decades due to its importance in physical interaction with robotic devices, for assistive, rehabilitation, and performance augmentation purposes. Humans can vary the dynamics of their interaction with a robot by changing the configuration of their limbs and/or modifying limb stiffness through co-contraction of opposing muscles (Perreault et al., 2001; Krebs et al., 2003; O’Neill et al., 2013; Patel et al., 2013). From a robotics point of view, Hogan (1985) showed that these dynamics can be dealt with by effectively utilizing impedance as a way of controlling the robot and its interactions with humans and external objects.

Human–robot interaction applications motivated the design of our study. Nevertheless, our results should be considered a preliminary step in the context of these potential applications due to the fact that our setup is a simplified version of tasks with more complex mechanics. Although the human–human interaction scenario we investigated is not representative of all contexts involving physical collaborations between two human agents, or human–robot agents, we believe that our work provides important insights about the feasibility of using impedance as a viable means of human–robot communication. Specifically, in a collaboration task similar to the one used for the present study, the robot arm controller could be trained to probe or sense—as the “leader” or “follower,” respectively—the impedance or exploratory movements of the human agent and assist his/her movement accordingly. Further work, however, is needed to quantify the extent to which such impedance-based controller can mimic the human–human co-adaptation described in the present study.

It is worth noting that several studies have investigated physical human–robot interaction (Duchaine and Gosselin, 2009; Lecours et al., 2012; Cherubini et al., 2013) and the use of impedance in human–robot interactions (e.g., Lin et al., 2013; Nisky et al., 2013; Quek et al., 2013). The key difference between prior work and the current study is that this work is the first investigation of humans’ ability to use stiffness as a means of communicating intended direction of motion. It should be emphasized that the intended movement direction was effectively communicated without generating significant motion. Thus, this result underscores humans’ ability to convey and to understand intended movement direction through the modulation of stiffness *in the absence of* or *before* an actual movement. Our approach points to applications where a human or robot follower can intuitively learn to recognize when or whether the movement direction of the leader may be incorrect or hazardous. Additionally, this approach can also be utilized as a two-way method of communication for ambiguous situations during cooperative tasks. As such, our work contributes to the insights provided by research in the area of human–human and human–machine physical interaction (Reed and Peshkin, 2008; Jarrasse et al., 2012; Ganesh et al., 2014; Sawers and Ting, 2014).

With respect to the time it took followers to infer the leader’s intended direction (~30 s), these latencies are too long in human–robot interaction scenarios where speed and safety are important criteria. Nevertheless, it is conceivable that the response latencies could be potentially reduced were participants to be exposed to a lower number of intended directions (e.g., four cardinal directions). Further work is needed to leverage our findings for human–robot interaction applications.

### Impedance-Based Communication of High-level Movement Goals

At least two theoretical frameworks—that differ in terms of whether a physical interaction between two agents is necessary or not—could account for our results. One of these frameworks would predict that humans modulate their arm stiffness as a function of planned movement in a given direction, regardless of whether another agent is probing their intended movement direction. If so, our findings would indicate that the follower learns how to capture the force–displacement relationship, which might imply the stiffness modulation to correctly infer the leader’s intended movement direction. However, an alternative framework would predict that the leader—consciously or sub-consciously—gradually learned that modulating arm stiffness was an effective or the best way to communicate his/her intended movement direction to the follower.

Our present data do not allow distinguishing between these two alternative frameworks. Therefore, future work is needed to determine the neural mechanisms responsible for non-verbal communication of movement direction through stiffness modulation and co-adaptation of two cooperating agents. Nevertheless, the fact that our dyads improved with practice in communicating and inferring movement direction would favor the second framework as the most plausible scenario. Future work will address the underlying neural mechanisms.

In the aspect of admittance/impedance relation to describe the coupled interaction (Hogan, 1985), the way the roles of leader and follower were defined may suggest that the leader must operate as an admittance (reading an input force and responding with a motion) and the follower as an impedance (apply a force and read a motion). The question is raised to what extent the two actors can strictly interpret the task in this sense, in which case the leader would in fact modulate admittance not impedance. However, another scenario could be that the follower applies probing motions (not forces), senses the leader’s resistive force, and observes the error caused by the leader’s resistance. As the follower’s task has a positional constraint (remaining within the 5-cm circle), it is more likely that the follower tries to perform a motion and senses a resistance, i.e., the follower is interpreting the leader as an impedance.

## Conclusion

We found that agents performing a collaborative manipulation task were able to non-verbally communicate/infer intended movement direction even when VF of arm configuration or handle was not available. With practice, the ability to correctly infer intended movement direction improved in parallel with a directionally tuned modulation of force–displacement relationship which might imply aspects of peoples’ modulation of arm stiffness/impedance. We conclude that human body (mainly upper limb) stiffness, extracted through haptic feedback alone, can be successfully used to infer/communicate intended movement direction. These results provide proof of concept for potential applications to human–robot interactions, where artificial controllers could be designed to capitalize on estimating and reacting to human limb stiffness.

## Author Contributions

Data collection: KM and BW. Design, analyzing data, and preparing manuscript and figures: all the authors.

## Conflict of Interest Statement

The authors declare that the research was conducted in the absence of any commercial or financial relationships that could be construed as a potential conflict of interest.
