# Supplementary material for: Communication and Inference of Intended Movement Direction during Human–Human Physical Interaction
Source: Front Neurorobot. 2017 Apr 13;11:21. doi: 10.3389/fnbot.2017.00021 (PMC5390012; doi:10.3389/fnbot.2017.00021)
Supplement: Supplementary file 1 [file Presentation_1.PDF]

Supplementary Material

Communication and Inference of Intended Movement Direction during Human-Human Physical Interaction

Keivan Mojtahedi[1], Bryan Whitsell[2], Panagiotis Artemiadis[2], Marco Santello[1*]

*** Correspondence:** Dr. Marco Santello: Marco.Santello@asu.edu

1 Average resultant force and maximum displacement analysis

The current study was designed to investigate whether and the extent to which the force-displacement relationship ($|k|$ or $|k|_{Normalized}$) can be used as a viable method of communicating intended movement direction. Based on the definition of $|k|$, movement direction associated with low $|k|$ would result in a larger displacement due to smaller force, and smaller displacement due to larger force for high $|k|$. Our focus on $|k|$ incorporates this relationship between force and displacement. Our interpretation about $|k|$ as a means of communication does not rule out, but rather includes the use of position sensing for this purpose. Specifically, we propose that position and force sensing combined were involved in the estimation of intended movement direction. We have provided **Supplementary Figure 1** to further illustrate this point. The maximum displacement at "movement direction with respect to intended direction" (denoted as "0" on the x-axis) is significantly different from all other directions (except ±1) across all trials. This result is consistent with $|k|$-based communication and implies that displacement alone did not lead to the correct inference of movement direction. Rather, this result suggests that subjects evaluated *both* the displacement and the amount of force that they applied across directions to infer the other subject's intended movement direction. For example, the inference subject made was associated with the exertion of larger forces in ±1 directions, which resulted in a smaller displacement in those directions compared to the 0 direction.

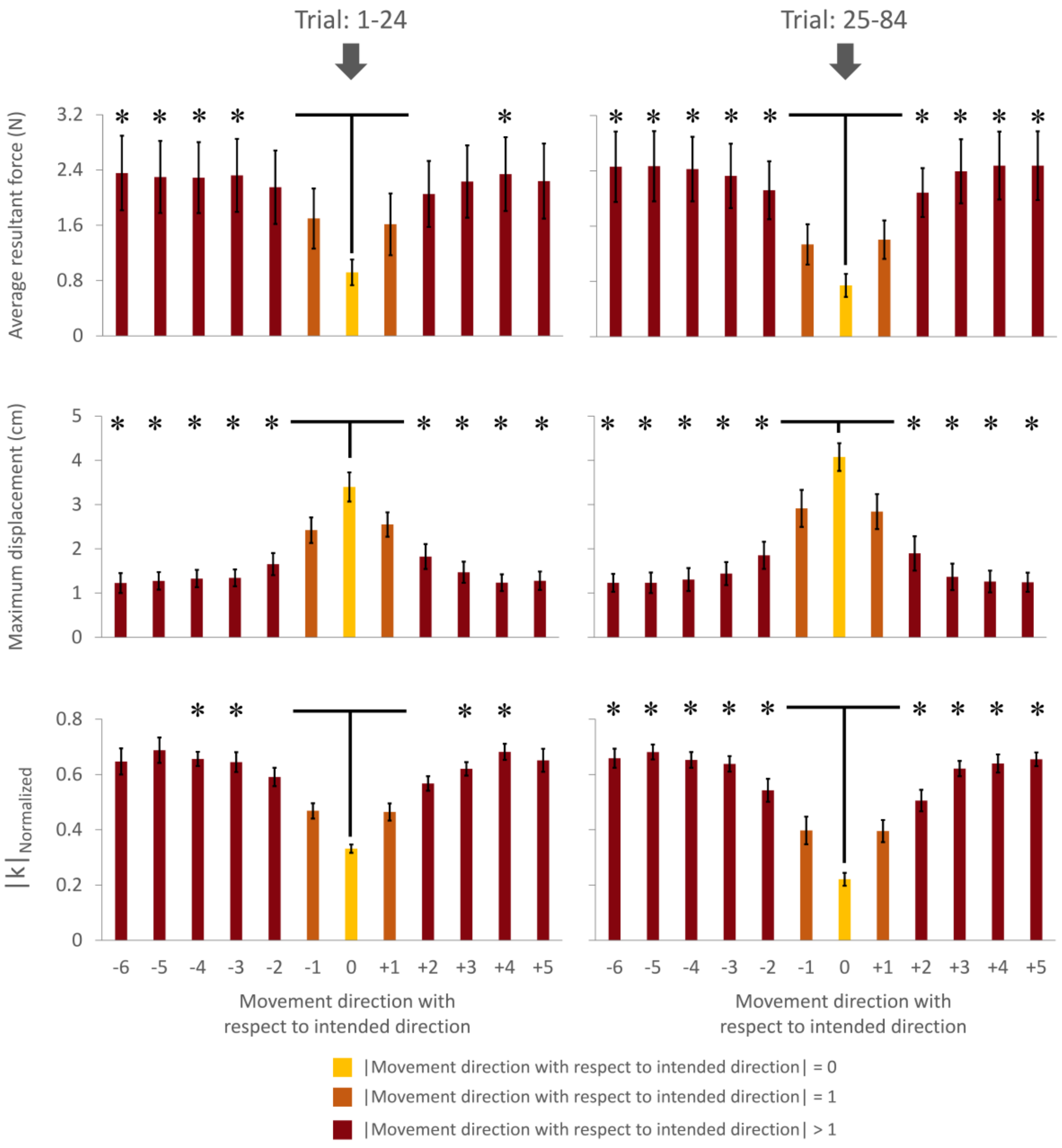

Supplementary Figure 1. Average resultant force, maximum displacement and the force-displacement relationship ($|k|_{Normalized}$) of group with visual feedback for each movement direction with respect to intended direction (all subjects; top to bottom plots). Trial: 1-24 and Trial: 25-84 are left and right columns. Asterisks indicate a statistically significant difference of pairwise comparison between 0 (yellow bar) and other (dark brown bar) movement direction with respect to intended direction ($p < 0.05$). Data are mean values averaged across all subjects. Vertical bars denote standard errors of the mean.

2 Logistic regression analysis: The force-displacement relationship is a better predictor of PAI than either force or displacement alone.

To capture the relationship between average resultant force, maximum displacement, and the force-displacement relationship versus error direction with respect to intended direction, we qualitatively examined box plots of average resultant force, maximum displacement, and the force-displacement relationship (**Supplementary Figure 2**). This examination revealed no clear one-to-one relation between any of these variables and percentage of accurate inference (PAI).

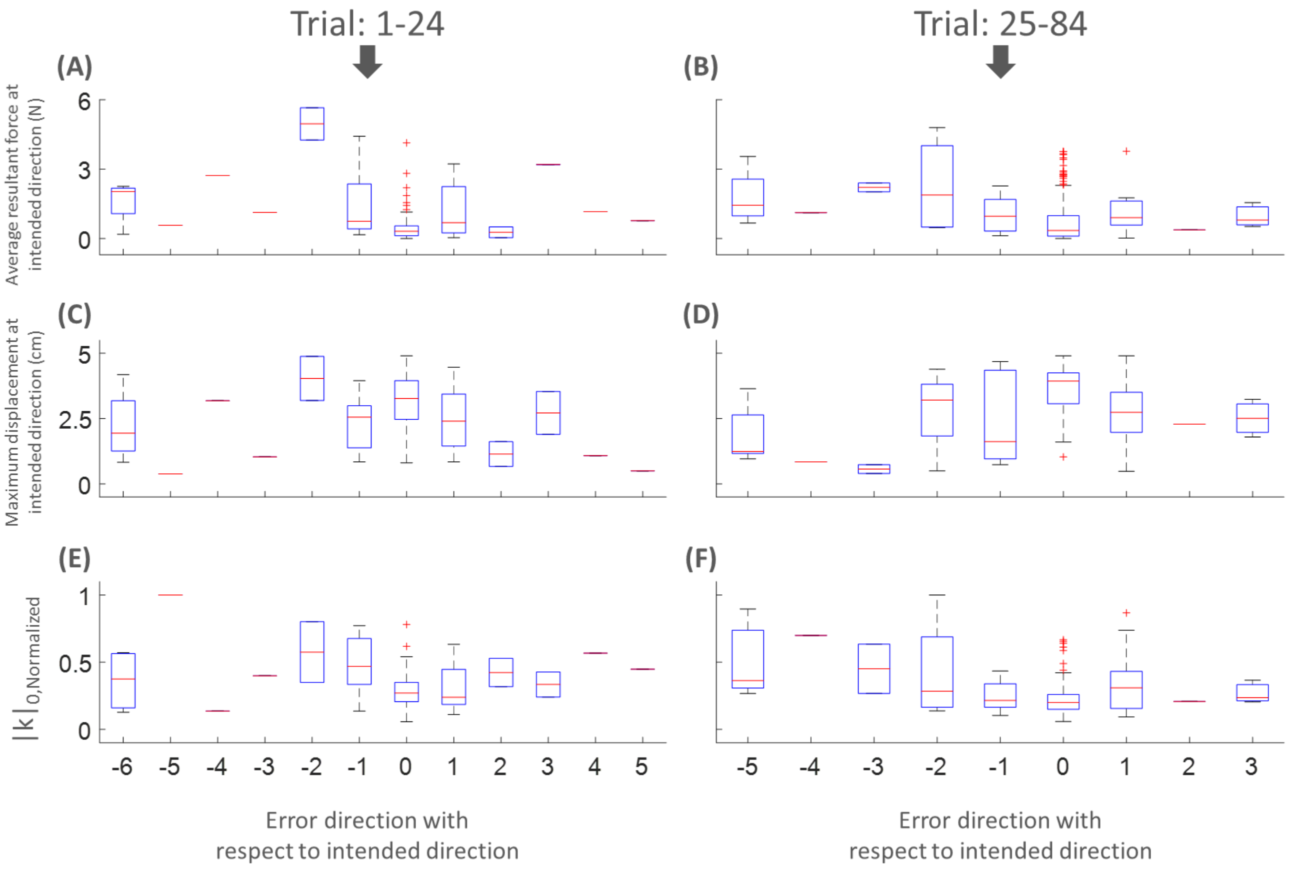

Supplementary Figure 2. Box-plots of average resultant force (A, B), maximum displacement (C, D) and the force-displacement relationship (E, F) of visual feedback group for each inferred movement direction with respect to intended direction (all subjects). Data from trials 1-24 and 25-84 are shown on the left and right columns, respectively. Data are mean values averaged across all subjects. Vertical bars denote standard errors of the mean.

As PAI consists of discrete values whereas the three above variables have continuous values, we applied logistic regression between PAI and each of the three variables by denoting 1 and 0 as the correct and wrong answers, respectively (regardless of the amount of error for wrong answers).

The plots in **Supplementary Figure 3** show the probability of participants' correct answer based on real data and fitted logistic function of the force-displacement relationship, average resultant force, and maximum displacement at intended direction for all subjects in the VF group. All the 15 logistic regressions (5 subjects × 3 fits, one per variable) were significant ($p<0.001$). To test which variable better predicts PAI, we calculated the correlation of estimated slope of logit function and intensity of

each variable. We found that the force-displacement relationship and force are better predictors of PAI than displacement. The R-values and p-values are slightly in favor of the force-displacement relationship than force, although the p-values are marginally insignificant. Importantly, there is an obvious difference between the slopes of fitted models of the force-displacement relationship and force. The estimated slopes of the force-displacement relationship is much larger than force which suggests that, with small differences in the force-displacement relationship, the probability of correct answers drastically changes (**F, Supplementary Figure 3**). In other words, subjects' correct answers are much more sensitive to changes in the force-displacement relationship than force and displacement. This may not be surprising, due to fact that the force-displacement relationship combines force and displacement. To sum up, the logistic regression analysis is in favor of the proposed notion that the force-displacement relationship is a better predictor of PAI than either average resultant force or maximum displacement alone at intended direction.

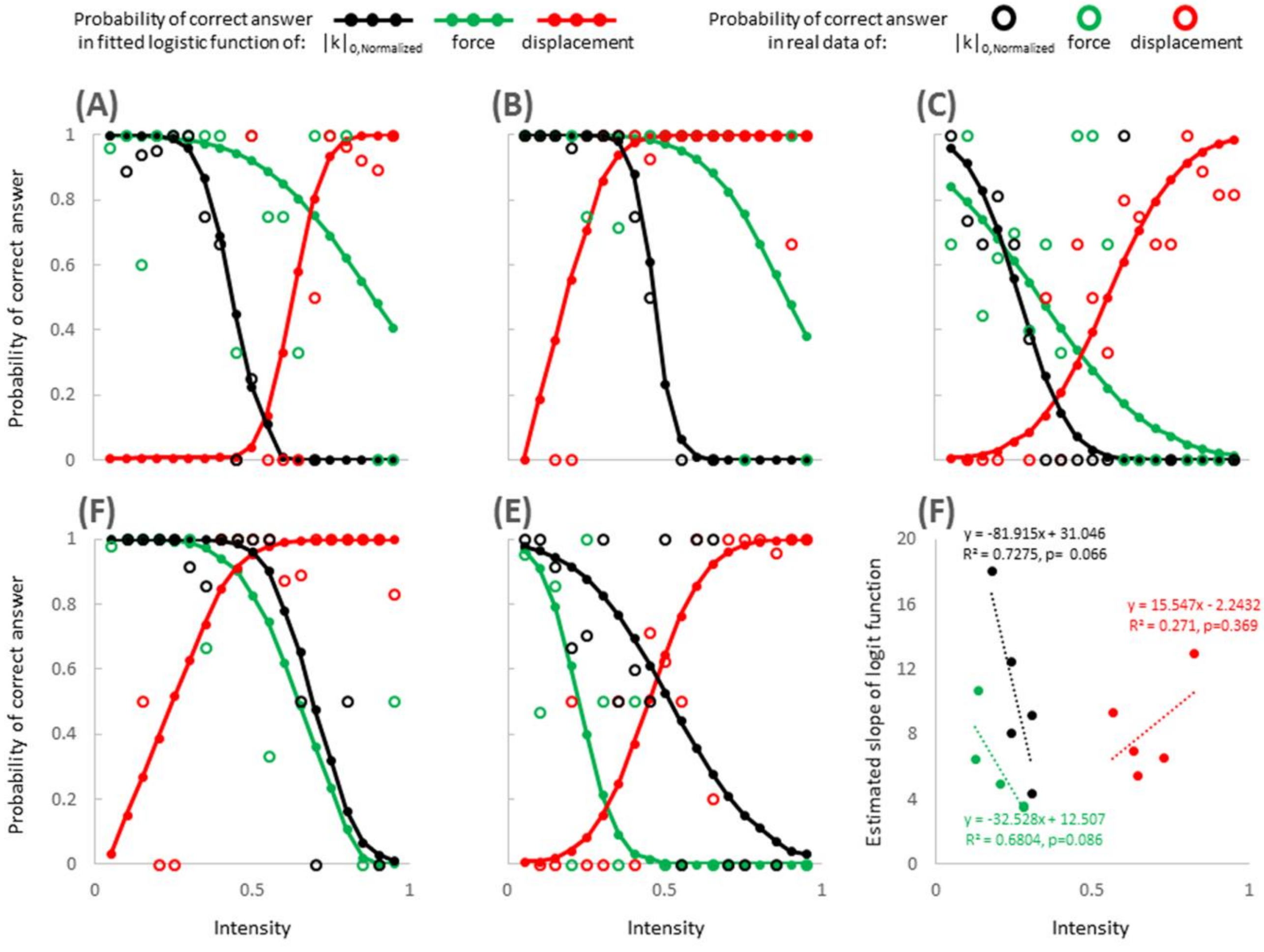

Supplementary Figure 3. Probability of participants' correct answer based on real data and fitted logistic function of the force-displacement relationship ($|k|_{0,\text{Normalized}}$), average resultant force, and maximum displacement at intended direction for all subjects in the VF group. The plots in (A) to (E) correspond to data from subject (or subject pair) 1 to 5, respectively. The empty and solid filled circles represent real data and data obtained through fitted logistic function, respectively. The black, green, and red colors denote the force-displacement relationship, average resultant force, maximum displacement; respectively. The lower intensities (magnitudes) of the force-displacement relationship and average resultant force at intended direction give

higher probability of participant's correct answer; whereas the higher intensities of maximum displacement lead to higher probability of correct answer. The plot in (F) shows the correlation between average intensity over 84 trials with the estimated slope of logit function for each variable. Note that the legend "force" and "displacement" correspond to average resultant force and maximum displacement at intended direction, respectively.

3 Force level in dyads is a very weak predictor of performance

To complement the above logistic regression analysis, we tested the relation between force not only at intended direction, but also at all directions, and PAI. **Supplementary Figure 4** shows very weak correlation between average resultant force magnitude across all directions and PAI. This results suggest that force is a very weak predictor of performance in our task.

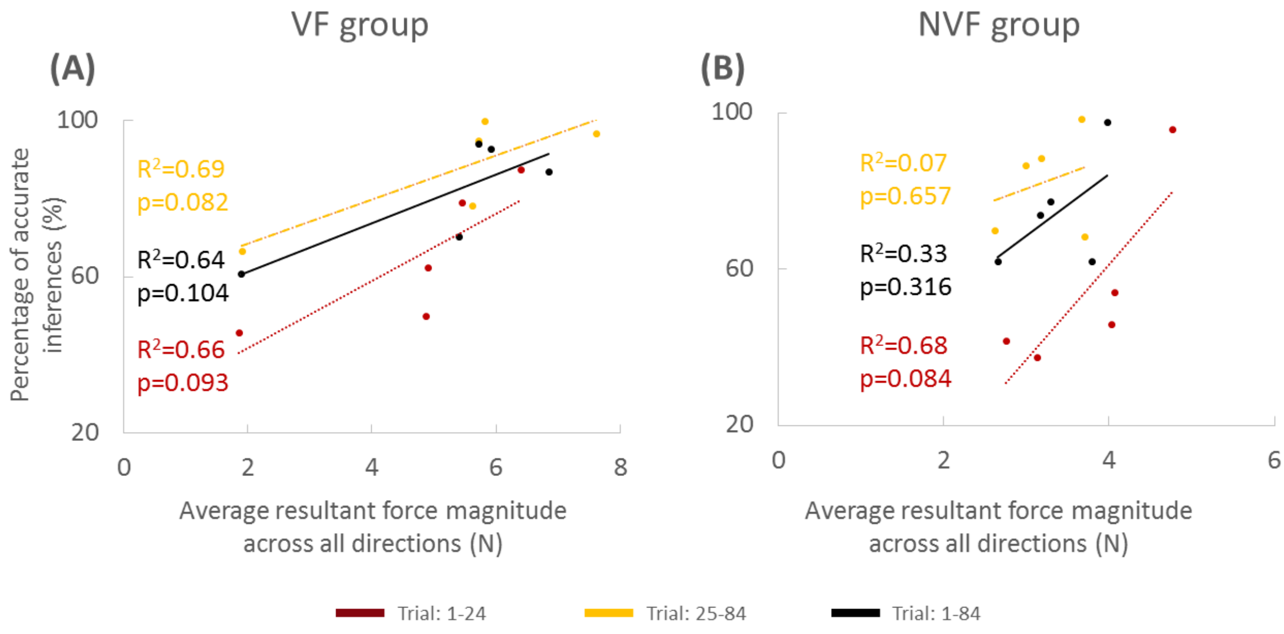

Supplementary Figure 4. Correlation between force magnitude and PAI across all subjects. Note that the force magnitude of each subject pair is obtained from averaging the resultant force across all directions.
